# Impact of Dementia on the Outcome of Surgical Revascularization for Chronic Limb-Threatening Ischemia

**DOI:** 10.3400/avd.oa.25-00040

**Published:** 2025-07-09

**Authors:** Atsushi Guntani, Shinsuke Mii, Kimihiro Komori

**Affiliations:** Department of Vascular Surgery, Saiseikai Yahata General Hospital, Kitakyushu, Fukuoka, Japan

**Keywords:** dementia, revascularization, chronic limb-threatening ischemia

## Abstract

**Objectives:** Dementia and chronic limb-threatening ischemia (CLTI) are independent risk factors for a poor life prognosis. We investigated the long-term results of surgical revascularization for CLTI complicated by dementia.

**Methods:** The clinical records of 174 consecutive patients with CLTI and 205 revascularized limbs were prospectively collected from a database. According to the criteria for dementia, the patients were divided into a low-grade dementia group (L group, n = 152) and a high-grade dementia group (H group, n = 22), and the long-term results after surgery were retrospectively analyzed.

**Results:** The 2-year amputation-free survival (AFS) after surgery was significantly lower in the H group than in the L group (L group, 82.3%; H group, 39.3%; p <0.001). However, no marked differences were observed between the dementia groups regarding the freedom from major adverse limb event (MALE) (L group, 86.6%; H group, 83.1%; p = 0.103), freedom from major adverse cardiovascular event (MACE) (L group, 75.6%; H group, 71.3%; p = 0.685), and limb salvage (L group, 75.6%; H, group 71.3%; p = 0.685) after surgery.

**Conclusions:** Dementia may be a predictor of a poor prognosis after surgery for CLTI. However, surgical revascularization may lead to limb salvage without serious postoperative complications. Therefore, surgical revascularization may be a useful treatment option if the patient or family requires such treatment.

## Introduction

The aging population is increasing worldwide, and the situation is particularly serious in Japan. Japan is an aging country with the highest prevalence of dementia worldwide. According to estimates, the number of people with dementia will exceed 7 million in Japan by 2025, and 1 in 5 people over 65 years old will develop dementia.^1)^ In the aging population, the number of patients suffering from both chronic limb-threatening ischemia (CLTI) and dementia is expected to increase.

Dementia and CLTI are reportedly independent factors of a poor life prognosis^2,3)^; however, few reports have evaluated the treatment outcomes of CLTI complicated with dementia.^4,5)^ Therefore, we investigated the long-term results of surgical revascularization for CLTI complicated with dementia and discussed the treatment options.

## Patients and Methods

### Patients

The clinical records of patients were prospectively collected from a database and retrospectively analyzed. We included 174 consecutive patients (205 limbs in which surgical revascularization was performed for CLTI) encountered between April 2015 and September 2021.

### The assessment of dementia

In all patients, the degree of dementia was classified by medical staff at the time of admission according to the criteria for determining the level of independence in daily life for elderly people with dementia established by the Japanese Ministry of Health and Welfare (available from: https://www.mhlw.go.jp/stf/seisakunitsuite/bunya/0000076236_00002.html.). The criteria were as follows: Grade I, having some sort of dementia but almost independent in daily domestic and social activities; Grade II, symptoms, behavior, or difficulty in communication that interferes with a person’s daily life are observed to some degree, but they can still live independently if someone looks in on them; Grade III, symptoms, behavior, or difficulty in communication that interfere with a person’s daily life are observed once in a while, and the patient requires care; Grade IV, symptoms, behavior, or difficulty in communication that interfere with the person’s daily life are observed frequently, and they require constant care; Grade M, significant psychotic manifestations, problem behavior, or severe physical diseases are observed, and the patient requires specialized medical care.

We divided the patients into 2 groups according to the criteria: Grade 0 (no dementia) and Grades I and II (observation-required dementia) were defined as the low-grade cognitive impairment group (L group), while Grades III and IV (assistance-required dementia) were defined as the high-grade cognitive impairment group (H group). Patients with Grade M were excluded because of the need for specialized medical care.

### Clinical outcome measures

The primary endpoint of this study was the 2-year amputation-free survival (AFS), and the secondary endpoints were the 2-year freedom from major adverse limb event (MALE), 2-year freedom from major adverse cardiovascular event (MACE), and 2-year limb salvage (LS) rate.

### Strategy for CLTI

We classified foot lesions according to the Society for Vascular Surgery Lower Extremity Threatened Limb Classification System, which involves risk stratification based on wound, ischemia, and foot infection (WIfI) classification,^6)^ and used the femoropopliteal (FP) and infrapopliteal (IP) grades to determine the Global Limb Anatomical Staging System (GLASS) stage.^7)^ We considered treatment strategies for CLTI, including evaluating the appropriateness of revascularization. Revascularization is contraindicated in patients who are bedridden and have contracted or extensive necrosis of the lower limbs. In such cases, or if the patient’s systemic condition is too severe to withstand invasive surgery, primary major amputation of the lower limb is recommended. We did not exclude patients with dementia from the indication for revascularization.

Our strategy for CLTI was to perform bypass surgery using an autologous vein as the first choice. However, when the lesion was within a short range, with Grades 0, I, and II in the FP or IP artery in the GLASS stage, or simple and small wounds according to the WIfI classification, endovascular treatment (EVT) was performed. In addition, EVT was performed when the systemic condition was too poor for bypass surgery, and bypass surgery using a prosthetic graft was performed in cases without appropriate autologous veins, especially in the FP artery region.

### The medications and follow-up

Postoperatively, unfractionated heparin was infused for 24 h to 7 days, and antiplatelet drugs were also administered to every patient and then permanently if not contraindicated. Anticoagulants using artificial grafts are generally prescribed to patients undergoing bypass surgery. However, the decision to use anticoagulants depended on the patient’s age, renal function, and general condition.

The patients were followed regularly at the outpatient clinic every 1–3 months for the first 2 years and 3–6 months thereafter. Routine surveillance included palpation of the arteries in the lower leg, ankle–brachial index, and duplex ultrasonography. Patients who could not visit the institution were interviewed on the phone at least annually to confirm their situation.

### Definitions

LS was defined as freedom from major amputation, which was defined as the loss of the leg above the ankle. MALE was defined as major amputation or major reintervention, including new bypass grafting, jump grafting, interposition graft revision, thrombectomy, or thrombolysis related to the indicated limb. MACE were defined as nonfatal myocardial infarction, nonfatal stroke, cardiovascular death, unstable angina, and heart failure. The AFS was defined as freedom from major amputation or death. In-hospital mortality was defined as all-cause mortality during hospitalization due to surgical revascularization.

### Statistical analyses

Categorical and continuous variables were compared using Fisher’s exact test and unpaired Student’s *t*-test, respectively. Survival curves were calculated using the Kaplan–Meier method and evaluated using the log-rank test. Propensity score matching was used to compare the AFS, MALE, MACE, and LS between the dementia groups. Statistical significance was set at p <0.05.

All statistical analyses were performed using the JMP Pro version 14.2.0 software program (SAS Institute, Cary, NC, USA).

## Results

### Baseline characteristics

The characteristics of the patients in this study are listed in **Table 1**. The patients included 102 men and 72 women, with a mean age of 75.5 years. The patients had a history of diabetes mellitus (59.8%), coronary artery disease (40.8%), cerebrovascular disease (33.3%), or end-stage renal disease (37.4%).

**Table:** Table 1 Patient characteristics

Factor	n = 174
Age (years), mean (range)	75.5 (46–100)
Female	72 (41.4%)
Diabetes mellitus	104 (59.8%)
Coronary artery disease	71 (40.8%)
Cerebrovascular disease	58 (33.3%)
End-stage renal disease	65 (37.4%)
Dyslipidemia	70 (40.5%)
Smoking history	97 (55.7%)
Aspirin use	78 (44.8%)
Clopidogrel use	73 (42.0%)
Cilostazol use	57 (32.8%)
Warfarin use	45 (25.9%)

A total of 152 (87.4%) patients were assigned to the L group and 22 (12.6%) to the H group, and 183 (89.3%) treated limbs were assigned to the L group and 22 (10.7%) to the H group according to the dementia assessment. The mean age was 74.6 years in the L group and 81.9 years in the H group, making the H group significantly older than the L group (p = 0.001). However, there were no significant differences between the groups regarding other factors.

### Details of revascularization for CLTI

All patients were symptomatic with threatened limb loss, and 42 (20.5%), 124 (60.5%), and 39 (19.0%) limbs were classified into Rutherford categories 4, 5, and 6, respectively. The number of limbs with WIfI stages 3 and 4 and GLASS stage 3 was 62 (33.5%), 88 (47.6%), and 172 (84.3%), respectively.

Revascularization of the common femoral artery, including endarterectomy of the common femoral artery and femorofemoral artery crossover bypass, was performed in 13 (6.3%) limbs. The target arteries for distal anastomoses in bypass surgery were the above-the-knee popliteal artery in 39 (19.0%), below-the-knee popliteal artery in 22 (10.7%), the tibial artery in 77 (37.6%), and the para-malleolar arteries in 54 (26.3%) limbs. In bypass surgery for 192 limbs, excluding 13 limbs with revascularization of the common femoral artery, autologous vein grafts were used in 162 limbs (84.4%), prosthetic vascular grafts were used in 22 limbs (11.5%), and composite grafts were used in 8 limbs (4.2%) (**Table 2**). There was no significant difference in the mean operation time (L group, 274.9 minutes; H group, 262.5 minutes; p = 0.4902) or mean amount of blood loss (L group, 254.6 mL; H group, 255.2 mL; p = 0.9926) between the groups.

**Table:** Table 2 Details of ischemic limbs and procedures

Factor	n = 205
Rutherford categories	
4	42 (20.5%)
5	124 (60.5%)
6	39 (19.0%)
WIfI stage	
3	62 (33.5%)
4	88 (47.6%)
GLASS stage	
3	172 (84.3%)
Target arteries	
Common femoral	13 (6.3%)
AK popliteal	39 (19.0%)
BK popliteal	22 (10.7%)
Tibial	77 (37.6%)
Para-malleolar	54 (26.3%)
Conduits	
Autologous vein grafts	162 (84.4%)
Prosthetic grafts	22 (11.5%)
Composite grafts	8 ( 4.2%)

AK: above the knee; BK: below the knee; GLASS: Global Limb Anatomical Staging System; WIfI: wound, ischemia, and foot infection

### Outcomes of survival and adverse event

The mean follow-up duration after surgical revascularization was 2.64 years (median, 2.32 years). In-hospital deaths occurred in 12 cases, including 3 surgical deaths. The causes of death were mainly pneumonia and urinary tract infection, and 3 cases were caused by cardiovascular events. In-hospital death occurred in 8 of 183 cases in the L group (4.4%) and in 4 of 22 cases in the H group (18.2%). In-hospital death occurred more frequently in the H group than in the L group (p = 0.028).

**Figure 1:**
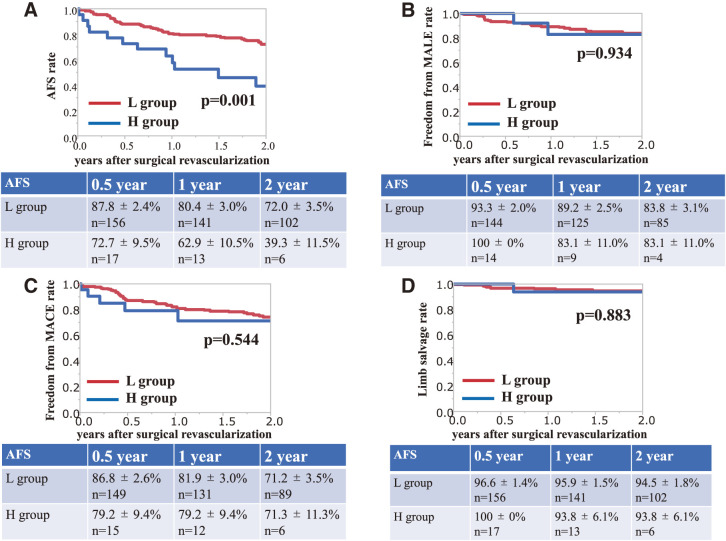
Fig. 1 (**A**) The amputation-free survival rate ± standard error after surgical revascularization. (**B**) The freedom from major adverse limb event rate ± standard error after surgical revascularization. (**C**) The freedom from major adverse cardiovascular event rate ± standard error after surgical revascularization. (**D**) The limb salvage rate ± standard error after surgical revascularization.

The 2-year AFS rate after surgical revascularization was significantly lower in the high-grade dementia group than in the low-grade group (L group, 72.0%; H group, 39.3%; p = 0.001) (**Fig. 1A**). However, no marked differences were observed between the dementia groups regarding the freedom from MALE rate (L group, 83.8%; H group, 83.1%; p = 0.934) (**Fig. 1B**), freedom from MACE rate (L group, 71.2%; H group, 71.3%; p = 0.544) (**Fig. 1C**), and LS rate (L group, 94.5%; H group, 93.8%; p = 0.883) (**Fig. 1D**) at 2 years after surgical revascularization.

**Figure 2:**
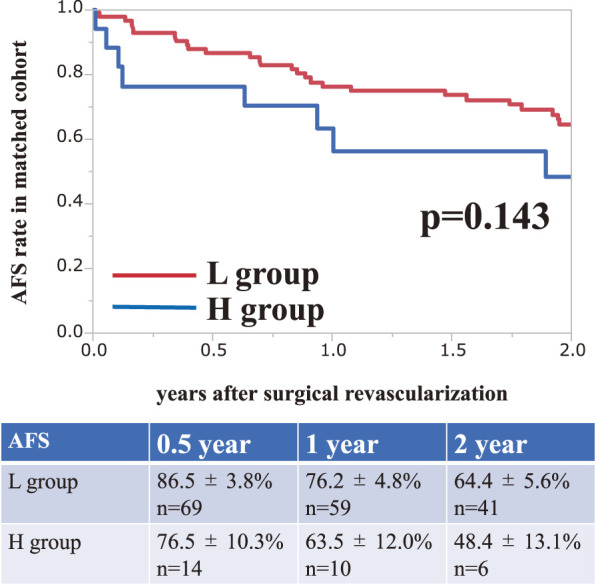
Fig. 2 The amputation-free survival rate ± standard error after surgical revascularization in the cohort matched for age.

As shown in previous results, the mean age in the H group was significantly higher than that in the L group. Therefore, we compared each group using propensity score matching for age. The mean age per limb was 74.6 years in the L group and 82.0 years in the H group (p = 0.001) in the unmatched cohort, and was adjusted to 79.6 years in the L group and 78.9 years in the H group (p = 0.853) in the matched cohort (**Table 3**). Although the H group showed a tendency toward a poor life prognosis, there was no significant difference between the groups (2-year AFS rate after surgical revascularization: L group, 64.4%; H group, 48.4%; p = 0.143) (**Fig. 2**).

**Table:** Table 3 Patient groups

Unmatched cohort			
	L group	H group	p Values
Limbs	183 (89.3%)	22 (10.7%)	
Mean age	74.6	82.0	0.001
**Matched cohort**			
	**L group**	**H group**	**p Values**
Limbs	83 (83.0%)	17 (17.0%)	
Mean age	79.6	78.9	0.853

## Discussion

The present findings suggest that dementia may be a predictor of a poor prognosis after surgical revascularization for CLTI; however, surgical revascularization, including major amputation, was identified as an important approach for avoiding MALE. Furthermore, dementia was not a predictor of a poor life prognosis after surgical revascularization for CLTI in the cohort matched for mean age. These results suggest that by salvaging the lower limbs, achieving wound healing, and relieving pain, it is possible for patients with dementia to maintain their activities of daily living (ADL), leading to an improved quality of life (QOL) for their remaining lifespan.

Previous reports have indicated that dementia is associated with poor outcomes, including higher complication rates, higher costs, increased 30- and 90-day mortality, and longer hospital stays in patients undergoing vascular surgery than those without it.^5,8)^ In addition, patients with CLTI have many comorbidities, such as diabetes, which are also risk factors for dementia.^9,10)^ Our patients were elderly and had a high incidence of comorbidities, such as diabetes, but other than age, there were no significant differences in baseline characteristics between the dementia groups.

While patients who undergo major amputation of the lower limbs due to CLTI have a marked decline in their ADL, it has been reported that patients who underwent revascularization were able to significantly improve their QOL in the long term. Primary amputation may be suggested for frail patients with CLTI, but amputation further worsens the patient’s ADL. As a patient’s ADL declines, the burden of care on the family also increases. This suggests that some patients with CLTI in clinical practice may be candidates for LS with revascularization.^8,11–14)^

Based on past literature and research results from our facility, bypass surgery is recommended as superior to catheter therapy in terms of ideal outcomes for patients who can tolerate surgical intervention.^7)^ Furthermore, as pointed out by Morisaki et al., bypass surgery is recommended for complex lesions classified as indeterminate and bypass-preferred category based on Global Vascular Guidelines.^7,14–17)^ Although EVT has the advantage of being minimally invasive, it is difficult to achieve wound healing and requires more additional treatment. If a patient can tolerate surgery, we believe that bypass surgery, which can be expected to have a definite therapeutic outcome, should be the 1st choice. In fact, the GLASS and WIfI classification of our cases showed a high proportion of complex lesions (GLASS stage 3: 84.3%; WIfI stage 3/4: 33.5%/47.6%) (**Table 2**), so we decided to select surgical revascularization.

We reviewed the details of in-hospital deaths to determine whether or not the decisions regarding surgical tolerability were appropriate. The most frequent cause of in-hospital death was postoperative pneumonia (41.7%). Causes of death other than cardiovascular events, such as pneumonia, accounted for 75.0% of such cases, which was observed in many elderly people regardless of dementia. The in-hospital mortality rate was significantly higher in the H group than that in the L group (L group, 4.4%; H group, 18.2%; p = 0.028). It was assumed that patients with dementia often have an impaired swallowing function; however, the reasons why postoperative pneumonia and in-hospital death are more common in patients with dementia than in those without it are unclear.

Finally, our findings suggest that the degree of dementia should be routinely assessed and considered in the management of elderly patients with CLTI, as it may affect not only their prognosis but also their ADL and QOL. Selecting an appropriate treatment method for CLTI requires a comprehensive assessment of the general condition, complexity of vascular lesions, and therapeutic goals desired by the patient and family. A larger series and longer follow-up will be needed to obtain more information and establish a standardized treatment method for CLTI patients with dementia.

### Study limitations

Several limitations of the present study warrant mention. First, this was a retrospective study, and the patient population was small. Second, this study is limited in its understanding of all CLTI patients with dementia, as patients who underwent EVT or primary major amputation were excluded.

## Conclusion

Dementia alone may not be a determining factor for surgical tolerability, but it was a predictor of a poor prognosis. Surgical revascularization for patients with CLTI and dementia is not a determinant of a poor prognosis and may be an important treatment option because it has the potential to maintain ADL and improve QOL. For patients with CLTI and dementia, treatment methods must be carefully selected with reference to the therapeutic goals of the patients and their families.

## Declarations

### Acknowledgments

Not applicable.

### Availability of data and materials

All datasets supporting the conclusions of this article are included in this published article.

### Ethics approval

This study was carried out in accordance with the principles of the Declaration of Helsinki. This project was approved by the Institutional Review Board of our facility (registration number: 190).

### Disclosure statement

The authors declare that they have no competing interests.

### Declaration of generative AI and AI-assisted technologies in the writing process

The authors did not use any generative AI and AI-assisted technologies.

### Author contributions

Study conception and analysis: AG, SM, KK

Data collection: AG, SM

Writing: AG

Critical review and revision: all authors

Final approval of the article: all authors

Accountability for all aspects of the work: all authors.
